# Multi-Spectral Imaging from an Unmanned Aerial Vehicle Enables the Assessment of Seasonal Leaf Area Dynamics of Sorghum Breeding Lines

**DOI:** 10.3389/fpls.2017.01532

**Published:** 2017-09-08

**Authors:** Andries B. Potgieter, Barbara George-Jaeggli, Scott C. Chapman, Kenneth Laws, Luz A. Suárez Cadavid, Jemima Wixted, James Watson, Mark Eldridge, David R. Jordan, Graeme L. Hammer

**Affiliations:** ^1^Queensland Alliance for Agriculture and Food Innovation, University of Queensland Toowoomba, QLD, Australia; ^2^Queensland Alliance for Agriculture and Food Innovation, University of Queensland Warwick, QLD, Australia; ^3^Agri-Science Queensland, Department of Agriculture and Fisheries Warwick, QLD, Australia; ^4^School of Agriculture and Food Sciences, University of Queensland Gatton, QLD, Australia; ^5^CSIRO Agriculture and Food St. Lucia, QLD, Australia; ^6^Queensland Alliance for Agriculture and Food Innovation, University of Queensland St. Lucia, QLD, Australia

**Keywords:** crop cover, mosaics, UAV, leaf area dynamics, water use, sorghum breeding

## Abstract

Genetic improvement in sorghum breeding programs requires the assessment of adaptation traits in small-plot breeding trials across multiple environments. Many of these phenotypic assessments are made by manual measurement or visual scoring, both of which are time consuming and expensive. This limits trial size and the potential for genetic gain. In addition, these methods are typically restricted to point estimates of particular traits, such as leaf senescence or flowering and do not capture the dynamic nature of crop growth. In water-limited environments in particular, information on leaf area development over time would provide valuable insight into water use and adaptation to water scarcity during specific phenological stages of crop development. Current methods to estimate plant leaf area index (LAI) involve destructive sampling and are not practical in breeding. Unmanned aerial vehicles (UAV) and proximal-sensing technologies open new opportunities to assess these traits multiple times in large small-plot trials. We analyzed vegetation-specific crop indices obtained from a narrowband multi-spectral camera on board a UAV platform flown over a small pilot trial with 30 plots (10 genotypes randomized within 3 blocks). Due to variable emergence we were able to assess the utility of these vegetation indices to estimate canopy cover and LAI over a large range of plant densities. We found good correlations between the Normalized Difference Vegetation Index (NDVI) and the Enhanced Vegetation Index (EVI) with plant number per plot, canopy cover and LAI both during the vegetative growth phase (pre-anthesis) and at maximum canopy cover shortly after anthesis. We also analyzed the utility of time-sequence data to assess the senescence pattern of sorghum genotypes known as fast (senescent) or slow senescing (stay-green) types. The Normalized Difference Red Edge (NDRE) index which estimates leaf chlorophyll content was most useful in characterizing the leaf area dynamics/senescence patterns of contrasting genotypes. These methods to monitor dynamics of green and senesced leaf area are suitable for out-scaling to enhance phenotyping of additional crop canopy characteristics and likely crop yield responses among genotypes across large fields and multiple dates.

## Introduction

Sorghum is the dominant dry-land summer crop in the north-eastern Australian grain belt. The growing environments of this area are characterized by high temperatures and variable rainfall, although many of the soils have sufficient water-holding capacity to allow crops to grow on stored sub-soil moisture (Pratley, 2003). As sub-soil moisture is depleted, mild or severe drought stress frequently develops toward the end of the growing season (Chapman et al., 2002), reducing crop yield. In the next decades, this situation is expected to occur even more frequently with increasing climate variability and weather patterns becoming more extreme (Lobell et al., 2015a) as was seen during the last two decades globally as well as in Australia (IPCC, 2014).

With changes in climate, quantitative breeding for specific traits that enhance yield in water-limited environments, will become even more important. One such trait is leaf area index (LAI), as the size of the crop canopy has important consequences for water use (Borrell et al., 2014a,b). Being able to accurately characterize leaf area would greatly enhance the selection of sorghum genotypes that are well adapted to water-limited environments. For example, in environments with mild to severe terminal drought stress, crops with smaller leaf area per plant have been found to have a yield advantage, as their reduced water use before flowering conserves sub-soil moisture that can be accessed during the critical grain-filling period (He et al., 2016). The stay-green trait in sorghum, which is associated with reduced leaf senescence and yield benefits under post-anthesis drought is thought to operate via this mechanism by conferring reduced tillering and smaller plant leaf areas before flowering (Borrell et al., 2014a,b). Stay-green has been an important trait in Australia's sorghum breeding programs, which has partly contributed to significant increases in sorghum yield trends in dry environments compared to moderate and wet environments over the last three decades (Potgieter et al., 2016). Up to now, breeders have positively selected for stay-green by visually rating leaf senescence after flowering. However, this only works in trials in which the right drought conditions develop for the trait to be expressed.

Apart from these links to evapotranspiration (George-Jaeggli et al., 2017), LAI is also useful to evaluate the fraction of absorbed photosynthetically active radiation, which is required to model canopy photosynthesis (Weiss et al., 2004). Being able to measure leaf area development over time would therefore allow the estimation not only of the water use pattern of a genotype, but also its likely photosynthetic output.

While visual scores of stay-green during grain-filling can be reasonably accurate when assessed at the right time and under the right level of water limitation, it is difficult to estimate plant leaf area or leaf area index (LAI) earlier in the season, and actual measurements of leaf area are time-consuming. Measurement of leaf area on thousands of plots at one time point, let alone several time points throughout the growing season is impractical. A low-cost high-throughput method for phenotyping canopy size of sorghum genotypes is needed.

The first application of remotely-sensed multi-spectral imagery and the development of vegetation indices to monitor crops goes back to the first NASA LANDSAT series in the 1970's (Tucker, 1979). The application of remote-sensing technology, in particular, hyperspectral imaging (Goetz, 2009), in vegetation mapping and yield forecasting has been steadily developing since then, and many different indices using specific wavelengths have been developed that can be used to assess plant growth parameters (Beeri and Peled, 2006; White et al., 2012). More recently this has been extended to predicting crop and vegetation biophysical attributes like net primary production (NPP), fraction of absorbed photosynthetically active radiation and LAI. This was done through the use of spectral indices (e.g., NDVI, EVI) derived from visible and near infrared reflectance spectra at moderate to high spatial resolutions across large scales (Huete et al., 2002; Hanes, 2014).

While the use of a digital camera attached to an Unmanned Aerial Device Unmanned aerial vehicles (UAV) was first proposed as a cost-effective way to monitor small wheat plots nearly a decade ago (Lelong et al., 2008) it was not until very recently that cheap, but highly precise positioning and digital imaging technologies and unmanned aerial device technology have become mainstream so that their use has become practical for farmers and research programs alike (Haboudane et al., 2004; Chapman et al., 2014; Candiago et al., 2015). The combination of these technologies provides the potential for high-throughput phenotyping to allow plant breeding programs to undertake quantitative screens of large breeding populations.

This paper presents results from a pilot study using a multi-rotor UAV fitted with a narrow-band multispectral camera (five bands of 10–40 nm width) to capture images of sorghum breeding lines with diverse canopy attributes across seven dates. We evaluated three narrow-band vegetation indices i.e., the normalized difference vegetation index (NDVI), the enhanced vegetation index (EVI) and the normalized difference red edge index (NDRE) to estimate traits, such as canopy cover, leaf area index and leaf chlorophyll content that are of particular interest to sorghum breeders in the northern grain belt of Australia.

Previous studies have demonstrated the utility of such vegetation indices to estimate LAI in soybean and maize (Viña et al., 2011) and wheat (Haghighattalab et al., 2016), but no such studies previously existed for sorghum. The objective of our study was to assess the suitability of vegetation indices calculated from spectral data captured with a multi-spectral camera mounted on a UAV to estimate canopy cover, leaf area and leaf chlorophyll content of a diverse set of sorghum genotypes grown in breeding plots. We also discuss the utility of such an approach to assess sorghum breeding lines for differences in canopy size and leaf chlorophyll content during critical crop stages, such as around flowering and during grain fill.

## Materials and methods

### Experiment and genotypes

An experiment was conducted to test the ability of multi-spectral sensing technologies on-board a UAV platform to calculate various vegetation indices to estimate canopy characteristics, such as plant cover, leaf area, leaf greenness or chlorophyll content and biomass of single plots sown to different sorghum genotypes. This paper only focuses on the outcomes related to plant cover, LAI and chlorophyll content.

Ten grain sorghum genotypes known for differences in canopy traits, such as plant height, leaf angle and leaf area were selected, including 4 genotypes with contrasting senescence type (i.e., rapid senescence after flowering = senescent type, or slow senescence = stay-green type).

**Figure 1:**
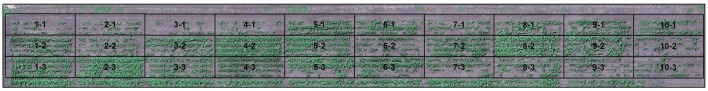
Experimental layout of plots at the Hermitage site as shown in aerial photo mosaic taken on 3 February. Plots were arranged in three rows and ten columns per row. Numbers in figure refer to the Column-Row position of each plot. Rows were treated as blocks and genotypes were completely randomized within each block (row).

The 10 sorghum genotypes were arranged in a randomized complete block design with 3 blocks (10 genotypes × 3 rows), resulting in 30 plots (Figure 1). Plots were 4 rows wide with 0.76 m row spacing by 10 meters long (i.e., 30.4 m^2^) and planted in an east-west direction.

The study site was located at the Hermitage Research Facility (28°12′ S, 152°06′ E; 480 m above sea level) in north-eastern Queensland. The soil of the trial area was conditioned 6 months prior to planting via incorporation of 3.5 t ha^−1^ of Gypsum, 350 kg ha^−1^ of NatraMin (AgSolutions, Australia) and 6 t ha^−1^ feedlot manure. One month prior to planting the trial area was fertilized with 220 kg ha^−1^ of GRAN-AM (20% Nitrogen, 24% Sulfur, Incitec Pivot, Australia) and 100 kg ha^−1^ of Urea (46% Nitrogen). The plots were sown with a precision planter on the 19th of November 2015. The trial was planted on a near-level site on a self-mulching alluvial clay with a high montmorillonite clay content (McKeown, 1978) that had a full sub-soil moisture profile at sowing. The trial was not irrigated, but regular in-crop rainfall and the sub-soil reserves prevented the development of significant water limitation. Crop establishment was variable due to surface flooding just after sowing. However, data are compared at the sample quadrat level (see details below) so that 30 sample quadrats at each harvest can essentially be considered as samples of potential leaf area for a diverse set of genotypes.

### Data capturing missions

At sowing time, accurate ground control points (GCP) were collected using a 1 cm resolution handheld GPS (Global Positioning System) unit (Trimble XT, Trimble, Sunnyvale California). Each of these GCPs was marked with a square concrete paver painted with blue triangles so that they were easily identifiable from above.

Data capturing missions were conducted at different critical times during the crop growth period (Table 1). Sample quadrat cuts of evenly established areas of two central rows (1 lineal meter from each) were taken within each plot at two different stages: pre-anthesis (ca. 8 weeks after sowing, or 3 weeks prior to anthesis) and at or within 1 week of anthesis.

**Table 1:** Experiment details, dates and variables collected.

**Experimental design**	**10 genotypes × 3 replicates (blocks), randomized complete block design**
Genotypes	R55637 (senescent), MR Buster (senescent), R931945-2-2 (stay-green), R931945-2-2TM (stay-green), 84G22, 85G56, FF_B963676, A1^*^F_B010054/F9_R04377-31, A1^*^F_B02055-9/R986087-2-4-1, R974443-1-2
UAV flights (2016)	12 January, 26 January, 3 February, 10 February, 16 February, 25 February, 31 March
Crop stages	Sowing—19 November 2015; Flowering—9 February 2016; Final Harvest—11 May 2016
Leaf area index (LAI) quadrat cuts	Pre-Anthesis (13 January) and Anthesis (9 February)
Number of culms per m^2^	Total number of culms (main stems and tillers together) at anthesis

*MR Buster and the two A1 genotypes are hybrids while the remaining genotypes are inbred lines*.

To reduce effects of ambient light condition, we limited data capturing missions to clear and cloudless days and conducted them around the middle of the morning.

### UAV platform

The UAV platform used was a 3D Robotics X8+ multi-rotor (Berkeley, California). The X8+ has the advantage of being able to fly at very low altitudes and at low speeds, which is critical for creating accurate and high-resolution mosaics (Corrigan, 2015). Flight altitudes for each flight were set at 20 m resulting in a ground sampling distance (GSD) or pixel size of ~0.5 cm.

### Multi-spectral camera

**Figure 2:**
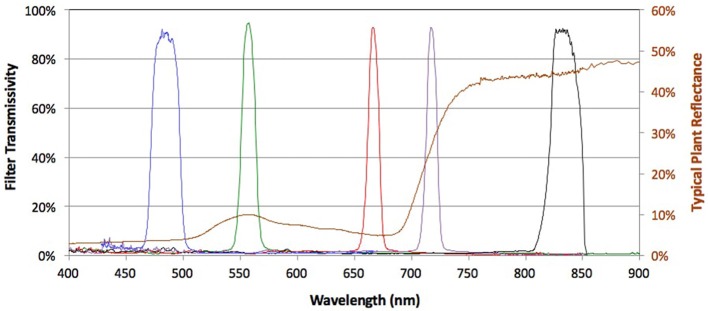
Multispectral bands of the Micasense™ camera across the spectrum of visible and infrared light. Peaks for each band's transmissivity are shown across the electromagnetic spectrum at specific nanometer (nm) wavelengths. Blue (B: 475 nm center wavelength, 20 nm bandwidth), Green (G: 560 nm, 20 nm), Red (R: 668 nm, 10 nm), Red Edge (RE: 717 nm, 10 nm), and Near Infrared (NIR: 840 nm, 40 nm). Source: http://www.micasense.com.

A RedEdge™ narrow-band multispectral camera (MicaSense, Seattle, Washington) (http://www.micasense.com/rededge/) simultaneously capturing 5 bands at specific nanometre (nm) wavelength peaks was fitted to the UAV platform. The bands captured were Blue (B: 475 nm center wavelength, 20 nm bandwidth), Green (G: 560 nm, 20 nm), Red (R: 668 nm, 10 nm), Red Edge (RE: 717 nm, 10 nm), and Near Infrared (NIR: 840 nm, 40 nm) (Figure 2). The camera captured the images and GPS information to a local digital card in 16-bit raw GeoTIFF files. This allowed for post geo-rectification and mosaicking. The horizontal field of view was 47.2 degrees with a 5.5 mm focal length producing an image resolution of 1,280 × 960 pixels.

The RedEdge camera includes factory calibration coefficients in each image for optics chain properties, such as lens distortion and optical vignetting. Atlas uses a CMOS sensor (Complementary metal–oxide–semiconductor) model along with extracted regions from images of a calibrated Lambertian reflectance panel to convert raw image digital number (DN) to reflectance units. These images are then linearly combined through a photogrammetry process to estimate the surface reflectance of each pixel in the final reflectance map.

### Mosaicking, ortho rectification and reflectance

After each flight, images for each of the five wavelengths were uploaded to the ATLAS cloud (MicaSense, Seattle, Washington) (http://www.micasense.com/atlas/). The cloud service uses the Pix4d software (PIX4d, Lausanne, Switzerland) (www.pix4d.com) and proprietary algorithms to stitch images together to create a geo-referenced multi-layer ortho-mosaic of the flight for each date. Stitched GEOTIFF format images for each band were downloaded from ATLAS and imported into ArcGIS (https://www.arcgis.com/home/index.html) for layer stacking and geo-rectification to GCP for each date.

To be able to convert DNs into reflectance, an image of a white reflectance panel was taken at the start and end of each flight and was uploaded with the images prior to the cloud processing. During the mosaicking process, the reflectance of the reference panel was used so that each of the 5 downloaded GeoTIFF files was a calibrated reflectance map for the respective band. The pixel values are proportional to % reflectance, with a pixel value of 32,768 being equal to 100% reflectance (65,535 is equal to 200% reflectance). Once the 5 bands had been layer stacked for a single flight date, data from each date was geo-rectified to high-precision GCP. Pixel values were then converted to reflectance values between 0 and 1 by dividing each pixel by the max reflectance value of 32,768. Pixels with specular reflectance (e.g., bright mirror like reflectance) and missing values from the mosaics were omitted in the analysis by masking.

After adjustment of pixel reflectance, indices per plot and per quadrat cut from each mosaic were generated in ArcGIS software and extracted and saved into an ASCII file format for comparison with measured data. The reflectance of areas of the quadrat cuts for any single flight could be determined by examining a subsequent flight (after cutting) to exactly identify where the cuts were made.

### Narrowband vegetation indices and percent cover

Two spectral indices were calculated from the reflectance measured by the RedEdge™ sensor. These indices relate to canopy health and canopy architecture (i.e., leaf area and biomass). The most widely used vegetation index is the Normalized Difference Vegetation Index (NDVI). NDVI is a simple normalized ratio between the NIR and R wavebands and is therefore a comparable metric between dates (Rouse et al., 1974):

(1) $$\begin{array}{r} \text{NDVI}=\left(\text{NIR}-\text{R}\right)/\left(\text{NIR}+\text{R}\right) \end{array}$$

We used a NDVI threshold of > 0.5 to capture reflectance from green leaves only and exclude soil background reflectance. This threshold has the greatest effect when plants are small i.e., at the time of the pre-anthesis measurement.

The enhanced vegetation index (EVI), which relates to canopy architecture was computed as follows (Huete et al., 2002):

(2) $$\begin{array}{r} \text{EVI}=2.5*\left[\frac{\text{NIR}-\text{R}}{\text{NIR}+6*\text{R}-7.5*\text{B}+1\;}\right] \end{array}$$

NDVI and EVI pixel values were aggregated to generate individual plot index metrics for each 30.4 m^2^ sorghum plot at each flight date. Maximum (NDVI_max_, EVI_max_) and average (NDVI_avg_, EVI_avg_) values for each index were derived from this time series.

In order to assess the degree of crop establishment (i.e., number of plants visible after emergence) we calculated the crop cover (CC, %) for each plot. An RGB image was generated from the Micasense mosaics. CC was then derived as the proportion of green pixels per plot. We defined pixels as “green” if their hue was between 70 and 140 degrees.

### Plant counts and leaf area index

Total number of plants per entire plot area (i.e., 30.4 m^2^) were counted 26 days after sowing (DAS). Leaf area (LAI) was measured destructively (sample quadrats) on the ground during the vegetative period (pre-anthesis, 54 DAS) and 2 weeks after the last genotype started flowering (anthesis, 83 DAS). At each sampling time, all plants within a 1.52 m^2^ sampling quadrat (2 × 1 m from the middle 2 rows of each 4-row plot), were cut at ground level and brought up to the laboratory for processing. Plants were separated into stems, leaves and panicles, main stems and tillers separately, and dried in a forced draft oven at 80°C until dry weight reached a minimum and then weighed. During the anthesis sample, culm numbers (main stem and tillers together) were recorded.

### Crop senescence

To analyze differences in rate of senescence between genotypes, we calculated the normalized difference red edge index (NDRE) (Gitelson and Merzlyak, 1994; Sims and Gamon, 2002):

(3) $$\begin{array}{r} \text{NDRE}=\left(\text{NIR}-\text{RE}\right)/\left(\text{NIR}+\text{RE}\right) \end{array}$$

The NDRE index is highly correlated with chlorophyll content within plants and therefore is a good surrogate for photosynthetic capacity (Gitelson and Merzlyak, 1994; Sims and Gamon, 2002; Gitelson et al., 2003). The difference between NDRE at maximum (peak) canopy cover and the NDRE at maturity (final flight date) was used as a simple metric for the rate of senescence (RS NDRE). To test whether this index was useful to differentiate between genotypes that were known to be senescent (tendency to senesce rapidly after flowering) or stay-green (tendency to senesce slowly after flowering), we grouped a subset of 4 genotypes into 2 groups (Senescent and Stay-green).

### Statistical validation metrics

All analyses and graphs were done using R (R Core Team, 2016).

We used simple linear regressions or logarithmic functions depending on best fit between vegetation indices (i.e., NDVI and EVI) and measured data at sample quadrat levels.

To test for significant genotype or group effects on individual vegetation indices we used linear mixed models in the lme4 package in R (Bates et al., 2015).

The general form of the mixed models used was:

(4) $$\begin{array}{r} \text{Y}=\text{X}\beta +\text{Z}\mu +\varepsilon \end{array}$$

where the response (vector y) is modeled by a set of fixed effects (vector β) and random effects (vector μ) and ε is the random error term. The design matrices X and Z assign the fixed and random effects, respectively to the observations.

For the time series of NDVI or NDRE vs. days after sowing (DAS) for individual genotypes, the mixed model included vector β comprising Genotype (factor with 10 levels) and DAS (factor with 7 levels) (fixed effects) and vector μ comprising Block (3 levels) within plot and vector ε comprising error (random effects).

To test whether Group (senescent or stay-green) had a significant effect on the difference between NDRE at maximum canopy cover and NDRE at maturity, we first tested a mixed model with vector β comprising Genotype (factor with 4 levels) and Group (factor with 2 levels), μ comprising Block (3 levels) and vector ε comprising error, but as Genotype had no significant effect, we only included Group in vector β in the final model. Assumptions of normality were tested with a quantile-quantile plot and seemed to have been met. Analysis of covariance was conducted with Group as variable to test whether the slopes of the relationship of NDRE vs. days after sowing during the post-anthesis period were significantly different between the senescent and stay-green genotypes.

## Results

### Vegetation indices aggregated at entire plot level

**Figure 3:**
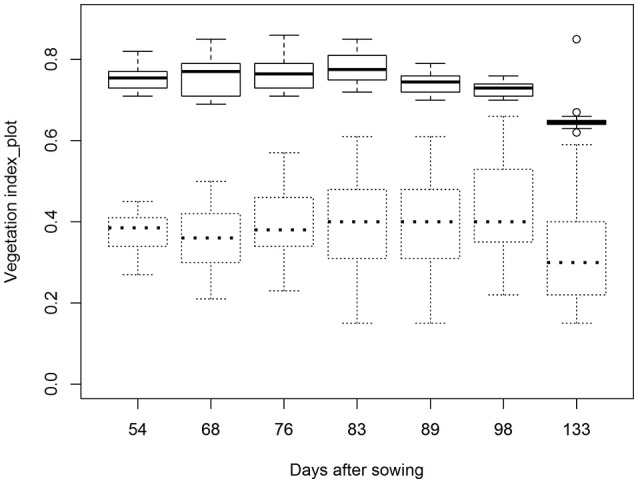
Boxplots showing NDVI (—) and EVI (—) values aggregated over entire 30.4 m^2^ plot areas and averaged across all 30 plots at seven flight dates. Flight dates were: 12 January or 54 days after sowing (DAS), 26 January (68 DAS), 3 February (76 DAS), 10 February (83 DAS), 16 February (89 DAS), 25 February (98 DAS), 31 March 2016 (133 DAS).

Averaged across all plots, NDVI (>0.5) and EVI values aggregated over the entire 30.4 m^2^ plot areas were 0.75 and 0.37, respectively, at the first flight date (Figure 3). Maximum values for NDVI occurred between 68 and 83 DAS and ranged from 0.72 to 0.86, depending on genotype. After this time, NDVI decreased due to crop senescence and reached values of between 0.62 and 0.67 by the end of the experiment (133 DAS) (Figure 3). Average EVI values remained relatively consistent for all, but the last flight date, when EVI was significantly lower (Figure 3).

### Percent cover at plot level

**Figure 4:**
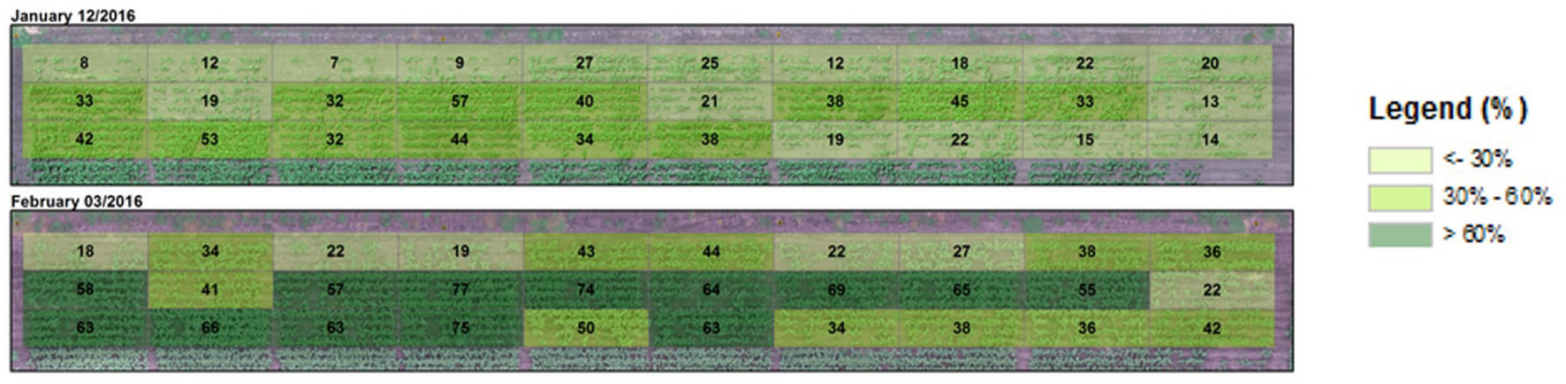
Aggregated crop cover calculated for entire plots super-imposed on a visible (narrow band RGB) image of the sorghum breeding experiment taken pre-anthesis (upper image) or a week before anthesis (lower). PC ranged from low (light green) to high (dark green).

**Figure 5:**
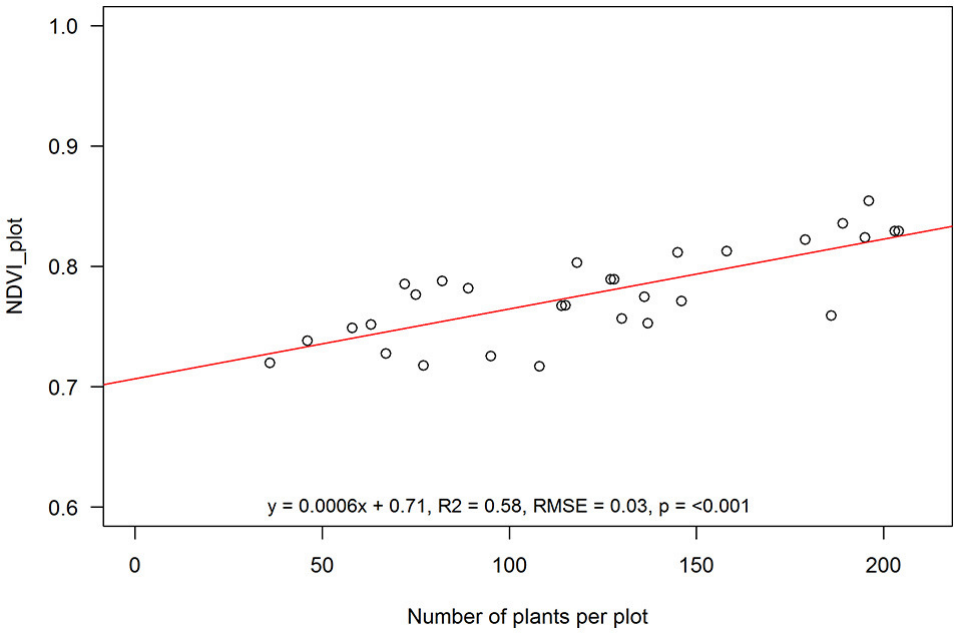
Aggregated NDVI for entire plots at anthesis against total plant number for each 30.4 m^2^ plot. Linear function: EVI = 0.0006^*^Plants + 0.71; R^2^: regression coefficient; RMSE, root mean square error; p, statistical significance level. Solid line is the fitted linear function through the sampling points (open circles).

Due to surface flooding in the first week after sowing affecting emergence, crop cover varied from as low as 7 and 18% to as high as 57 and 77% 54 days after sowing (DAS) (12 January) and 76 DAS (3 February), respectively (Figure 4). Actual plant counts ranged from 36 to 204 plants per plot (30.4 m^2^). This variability gave us an opportunity to test the validity of using NDVI to estimate crop cover across a broad range of plant covers. When aggregated over the entire plot area, NDVI (>0.5) was significantly and strongly correlated with plants per plot (*R*^2^ = 0.58, RMSE = 0.03, Figure 5).

Maximum crop cover at entire plot level was mostly >30 and 50% at the pre-anthesis (January image) and anthesis stages (February image), respectively (Figure 4).

### Correlation of vegetation indices with LAI measured by sampling quadrats

Leaf area index (LAI) values derived from the quadrat cut sampling areas ranged from 0.71 to 4.01 (m^2^/m^2^) at pre-flowering and increased to between 1.31 and 4.71 2 weeks after flowering in all plots.

Vegetation indices derived from pixels aggregated over the entire plot area at both the pre-flowering and the anthesis sampling dates were strongly linearly correlated with LAI from quadrat cuts (Table 2). The correlations were better for NDVI than for EVI as can be seen from greater regression coefficients (R^2^) and smaller root mean square errors (RMSE). NDVI_max_ also correlated well with LAI at the anthesis sampling date.

**Table 2:** Relationships between NDVI (>0.5) and EVI aggregated over entire plots (plot; 30.4 m^2^) or only the quadrat sampling areas (quadrat; 1.5 m^2^) with leaf area index (LAI) at 54 (pre-anthesis) and 83 (anthesis) days after sowing.

**Stage**	**Pixel aggregation level**	**Formula**	***R*^2^**	**RMSE**	***P*-value**
Pre-anthesis	NDVI_plot	NDVI = 0.025 ^*^ LAI + 0.703	0.55	0.019	<0.001
	NDVI_quadrat	NDVI = 0.034 ^*^ LAI + 0.690	0.85	0.011	<0.001
	EVI_plot	EVI = 0.027 ^*^ LAI + 0.316	0.19	0.045	<0.05
	EVI_quad	EVI = 0.139 ^*^ LAI + 0.138	0.81	0.056	<0.001
Anthesis	NDVI_plot	NDVI = 0.037 ^*^ LAI + 0.679	0.59	0.024	<0.001
	NDVI_max__plot	NDVI = 0.035 ^*^ LAI + 0.685	0.56	0.025	<0.001
	NDVI_quadrat	NDVI = 0.050 ^*^ LAI + 0.664	0.66	0.053	<0.001
	EVI_plot	EVI = 0.089 ^*^ LAI + 0.153	0.33	0.099	<0.001
	EVI_quadrat	EVI = 0.110^*^ LAI + 0.360	0.70	0.247	<0.001

*R^2^, Regression Coefficient; RMSE, Root Mean Square Error*.

When just aggregating the pixels over the actual sample quadrat areas the vegetation indices explained more of the variation in LAI as indicated by larger R^2^, but the RMSE did not always improve (Table 2).

**Figure 6:**
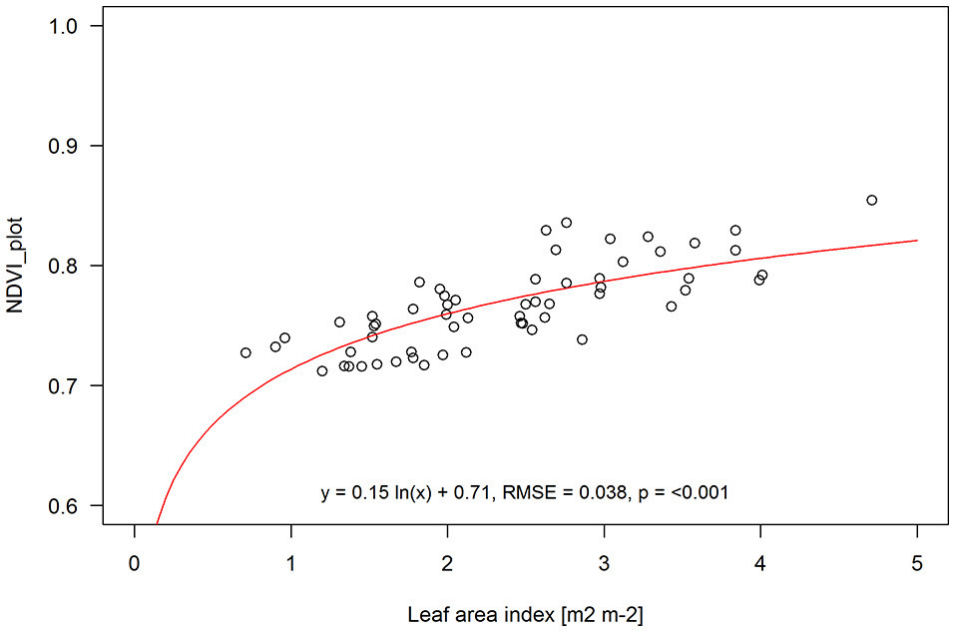
Aggregated NDVI for entire plots vs. LAI from quadrat cuts within each plot at both the pre-anthesis and anthesis sampling dates combined. Logarithmic function NDVI = 0.15 ln (x) + 0.71; R2: regression coefficient; RMSE, root mean square error; p, statistical significance level. Solid line is the fitted function through the sampling points (open circles).

When leaf area index data from quadrat cuts and NDVI aggregated over the entire plots was combined for both pre-anthesis and anthesis sampling dates (60 samples in total) a logarithmic function fitted the data slightly better than a linear one (RMSE of 0.038 vs. 0.041 for logarithmic and linear, respectively) (Figure 6).

### Temporal dynamics of NDVI and NDRE

**Figure 7:**
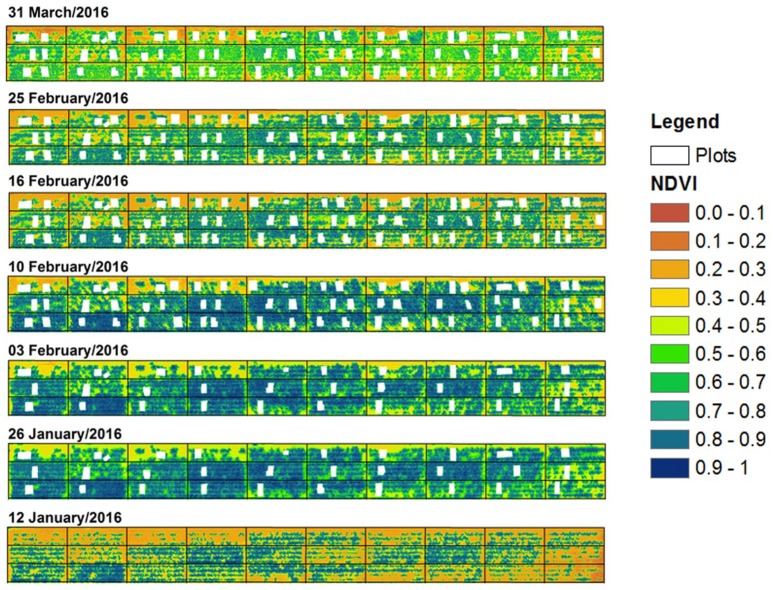
Normalized difference vegetation index (NDVI) for each date across the study area during the main growing period. Black dividing lines indicate plot boundaries, while white mask-out areas represent the areas where sample quadrat cuts were taken.

Normalized Difference Vegetation Index (NDVI) (aggregated over the entire plot) gradually increased and reached maximum values (>0.9; blue colored) by the anthesis sampling date (83 DAS or 10th February) (Figure 7). After this date, NDVI values decreased to around 0.5 (light green) due to progressive senescence of leaves as genotypes approached maturity.

**Figure 8:**
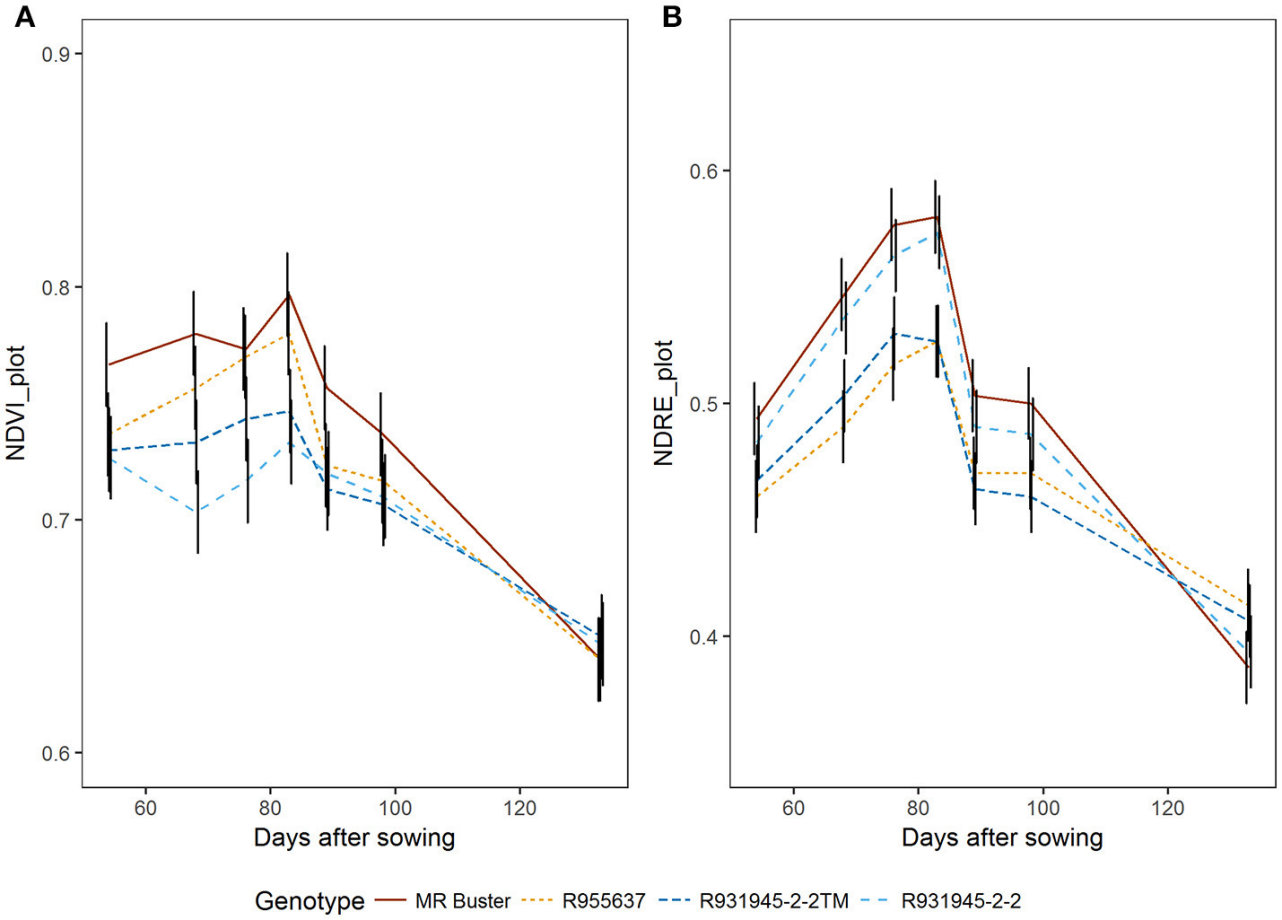
NDVI_avg_ **(A)** and NDRE_avg_ **(B)** aggregated over entire plot area from flowering to maturity for four sorghum genotypes contrasting in stay-green characteristics. MR Buster (brown) and R955637 (yellow) are both senescent types, while R931945-2-2 (light blue) and R931945-2-2TM (dark blue) are lines with the stay-green trait. Points are least squares means for NDVI and NDRE, respectively, predicted by the linear mixed model. Black vertical bars represent standard errors for three replicates at each time point.

NDRE values were much lower, but showed a similar pattern of slowly increasing up to about 2 weeks after flowering (83 DAS) and then decreasing as NDVI (Figure 8). As NDRE is related to chlorophyll, differences in NDRE values from peak canopy NDVI (i.e., NDVI_max_) to maturity (last flight date) are associated with the rate of senescence. The trial included genotypes that are known to senesce quickly (senescent genotypes; MR Buster and R955637) and others that have the stay-green trait meaning they have a slower rate or senescence and retain more green leaf area during grain fill compared with senescent types, particularly when water is limited (stay-green genotypes; R931945-2-2 and R931945-2-2TM) (Figure 8).

**Figure 9:**
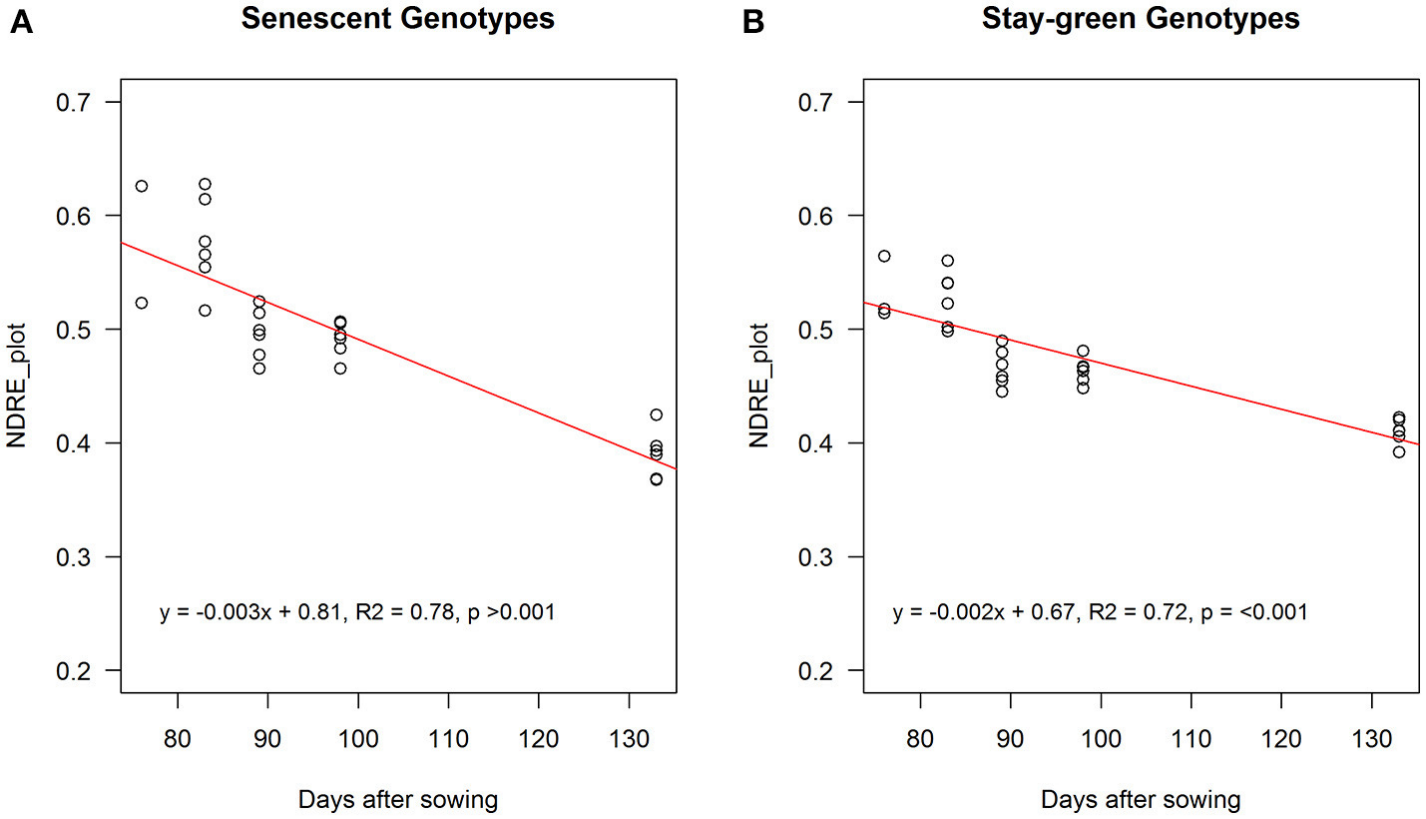
NDRE_avg_ aggregated over entire plot from maximum canopy cover to maturity (final flight) for senescent (MR Buster and R955637; **A**) and stay-green genotypes (R931945-2-2 and R931945-2-2TM; **B**). Points are values for individual plots. Solid lines are the fitted functions through the sampling points (open circles).

When these genotypes were grouped (i.e., stay-green group: R931945-2-2 and R931945-2-2TM and senescent group: MR Buster and R955637), RS NDRE (the difference between NDRE at maximum canopy cover and NDRE at harvest maturity), was significantly greater for the senescent group (0.19 vs. 0.13, *p* < 0.05, *n* = 12), indicating that these genotypes senesced at a faster rate compared with genotypes classified as stay-green genotypes. Consistent with this, the slope of the relationship between NDRE and DAS from maximum NDRE until maturity, was significantly steeper (−0.003 units per day) for the senescent group, compared with the stay-green group (−0.002 units per day) *p* < 0.01, indicating that the senescent genotypes lost chlorophyll significantly faster than the stay-green genotypes (Figure 9).

## Discussion

Due to variable plant numbers among plots in this pilot study, we were able to test the suitability of the vegetation indices to estimate percent cover and LAI over a range of densities. Actual plant counts per 30.4 m plot ranged from 36 to 204 plants. Correlations between actual plant number and percent cover estimated using NDVI were significant and moderately strong. The goodness of fit increased when masking was applied and pixels with NDVI lower than 0.5 (i.e., soil and non-living materials) were disregarded.

Correlations between vegetation indices and quadrat-estimated LAI improved when index values were aggregated over the sample quadrat area only rather than the entire plot area. Aggregating over the quadrat area alone provided a more direct correlation between the index and LAI, avoiding heterogeneity in canopy cover across the plot, associated with variable establishment. Sample cuts were selected where plant cover was more homogenous and therefore plot-level cover and derived vegetation index values differed from values derived from just sampling cut areas. Hence it is reasonable to assume that in more uniform trials, the expected relationship should be similar to that found for the quadrat comparisons (Table 2). Further experiments are being undertaken to confirm these relationships.

Likely limitations could exist when out-scaling this approach to other locations and crops. For example, the threshold used showed significant improvement in statistical analysis, similar to that of the EVI metric, but its utility requires further investigation. In addition, capturing data multiple times during the pilot study demonstrates the potential of these methods to study canopy dynamics. A likely constraint of comparing indices from different dates is that ambient light conditions may vary between flights. This was limited here by flying only on clear days with no clouds and during the middle of the morning. Furthermore, NDVI is less sensitive to such changes since it is a ratio index.

Previous studies have reported a saturation of NDVI at higher LAI values (i.e., LAI > 4) and thus in dense vegetation canopies using EVI might be preferable to NDVI (Huete et al., 2002; Myneni et al., 2002). Our experimental plots were all planted at a target population density of 5 plants per square meter and LAI at anthesis ranged from 1.3 to 4.7. When combining pre-anthesis and anthesis data we also observed a slight improvement in prediction power when fitting a logarithmic instead of a linear function (Figure 6). To assess LAI in sorghum breeding plots with higher LAI, it might also be better to use EVI instead of NDVI.

Peak NDVI values varied from 0.72 to 0.86 and end NDVI values from 0.62 to 0.67. In this trial, the end values were not greatly lower than maximum values, given that drought stress was not substantial, with plot yields in the uniform plots being over 9 t ha^−1^. Lines with the stay-green trait, R931945-2-2 and R931945-2-2TM, had a slower decline in NDRE after anthesis, compared with the two senescent genotypes, MR Buster and R955637. The stay-green trait has been associated with increased yield under post-anthesis drought (Borrell et al., 1999, 2000; Jordan et al., 2012) and due to the frequency of post-anthesis drought in sorghum growing areas, it has been actively selected for in Australian sorghum breeding programs. Being able to monitor senescence over time will assist breeders in selecting for stay-green under drought.

Breeding for yield under water-limitation has been the focus of sorghum breeding activities in Australia for the last three decades. This may well explain why sorghum yield advances in dry environments are currently more than double those in wet environments (Potgieter et al., 2016). However, there is potential to further improve yields in water-limited environments by improving the matching of leaf area and water-use dynamics to the temporal characteristics of drought (Chapman et al., 2000). The approach presented here offers the opportunity to monitor LAI of different genotypes throughout the crop-growing season, thus providing breeders with information on canopy dynamics. This will support the accelerated development and release of commercial hybrids that are matched to specific environments types.

In addition to plant breeders, agronomists and growers will also benefit from having access to information on crop canopy dynamics as it will allow them to estimate water use and expected yields for their sorghum crops as the season unfolds. Besides directly affecting crop water use (George-Jaeggli et al., 2017), LAI also relates to the fraction of absorbed photosynthetically active radiation (PAR) and therefore is one of the most important canopy attributes (Weiss et al., 2004; Sadras and McDonald, 2012; Sibley et al., 2014; Sadras and Calderini, 2015). LAI is an important input variable for crop models, such as APSIM (Keating et al., 2003) that are used for yield predictions at field and regional scales (Lobell et al., 2015b). An improvement in this methodology would be to be able to monitor the LAI as it increases toward a maximum value, and by accurately accounting for heads, soil and senescing leaves, to estimate the LAI as it changes during grain filling. A full-season measurement of LAI would allow use of these crop models in the estimation of seasonal crop growth and potential water use.

Apart from the capacity to scale phenotyping up from a few to thousands of breeders' plots, the approach presented here will facilitate the scaling-out of phenotyping from plant to plot to field scales and thus enabling industry to maximize yield potential at both the genetic and the agronomic level.

## Author contributions

AP and BG contributed equally to this manuscript. GH and DJ obtained funding for the study. AP, BG, SC, DJ, and GH planned and designed the experiments. BG organized the data collection and JWi, ME, and KL collected the on-ground and aerial data, respectively. AP, LS, JWa, and KL processed the images. BG and AP performed the statistical analyses, interpreted the results and drafted the manuscript with contributions from SC. All authors have read and approved the final manuscript.

### Conflict of interest statement

The authors declare that the research was conducted in the absence of any commercial or financial relationships that could be construed as a potential conflict of interest.
